# Enhanced Production of C_30_ Carotenoid 4,4'-Diaponeurosporene by Optimizing Culture Conditions of *Lactiplantibacillus plantarum* subsp. *plantarum* KCCP11226^T^

**DOI:** 10.4014/jmb.2204.04035

**Published:** 2022-05-31

**Authors:** Inonge Noni Siziya, Deok Jun Yoon, Mibang Kim, Myung-Ji Seo

**Affiliations:** 1 Division of Bioengineering, Incheon National University, Incheon 22012, Republic of Korea; 2 Research Center for Bio Materials & Process Development, Incheon National University, Incheon 22012, Republic of Korea; 3 Department of Bioengineering and Nano-Bioengineering, Incheon National University, Incheon 22012, Republic of Korea; 4 Department of Chemical Engineering, Pohang University of Science and Technology, Pohang, Gyeongbuk 37673, Republic of Korea

**Keywords:** Carotenoid, 4,4'-diaponeurosporene, *Lactiplantibacillus plantarum* subsp. *plantarum*, optimization

## Abstract

The rising demand for carotenoids can be met by microbial biosynthesis as a promising alternative to chemical synthesis and plant extraction. Several species of lactic acid bacteria (LAB) specifically produce C_30_ carotenoids and offer the added probiotic benefit of improved gut health and protection against chronic conditions. In this study, the recently characterized *Lactiplantibacillus plantarum* subsp. *plantarum* KCCP11226^T^ produced the rare C_30_ carotenoid, 4,4'-diaponeurosporene, and its yield was optimized for industrial production. The one-factor-at-a-time (OFAT) method was used to screen carbon and nitrogen sources, while the abiotic stresses of temperature, pH, and salinity, were evaluated for their effects on 4,4'-diaponeurosporene production. Lactose and beef extract were ideal for optimal carotenoid production at 25°C incubation in pH 7.0 medium with no salt. The main factors influencing 4,4'-diaponeurosporene yields, namely lactose level, beef extract concentration and initial pH, were enhanced using the Box-Behnken design under response surface methodology (RSM). Compared to commercial MRS medium, there was a 3.3-fold increase in carotenoid production in the optimized conditions of 15% lactose, 8.3% beef extract and initial pH of 6.9, producing a 4,4'-diaponeurosporene concentration of 0.033 A_470_/ml. To substantiate upscaling for industrial application, the optimal aeration rate in a 5 L fermentor was 0.3 vvm. This resulted in a further 3.8-fold increase in 4,4'-diaponeurosporene production, with a concentration of 0.042 A_470_/ml, compared to the flask-scale cultivation in commercial MRS medium. The present work confirms the optimization and scale-up feasibility of enhanced 4,4'-diaponeurosporene production by *L. plantarum* subsp. *plantarum* KCCP11226^T^.

## Introduction

Carotenoids are a broad class of organic pigments within the subfamily of lipophilic isoprenoids that can be found in both photosynthetic and non-photosynthetic organisms [1]. Although they can be isolated from various sources, only photosynthetic plants, algae, and certain types of bacteria and fungi can produce carotenoids through biosynthetic pathways. Animals, including humans, are non-carotenogenic organisms that acquire them from dietary sources [2]. Carotenoids tend to naturally exhibit colors ranging from yellowish to reddish hues, and differ in physical, chemical, and functional properties. These compounds are in increasing demand as they are widely used as additives in the food, feed, nutraceutical, cosmetic, and pharmacological industries. Their uses include biofortification, coloring, immunomodulation, chronic disease risk reduction and prevention of degenerative conditions [2]. Although commercial carotenoids are mainly obtained either by extraction from plants or chemical synthesis, the former method has the disadvantage of seasonal and geographic limitations, and the latter has negative effects on the environment due to byproduct generation, harsh production conditions, and hazardous waste. Microbial production is therefore preferable from both an economic and an ecological standpoint [3].

Carotenoids are typically composed of six, eight, nine and ten C_5_ isoprenoid units, and depending on the number of carbons, they can be categorized as C_30_, C_40_, C_45_ and C_50_ carotenoids, respectively. Among these, C_40_ carotenoids are abundant in nature and found mainly in plants and algae while C_30_ and C_50_ carotenoids are typically produced by bacteria and archaea [4]. In bacteria, carotenoids are located in the cell membrane as lipophilic compounds but their orientation depends on their structure and cell membrane thickness, which in turn affects extraction and yield of the synthesized products [5]. *Corynebacterium glutamicum* can produce the C_50_ carotenoid decaprenoxanthin and its C_45_ biosynthetic intermediate, nonaflavuxanthin, while *Gordonia alkanivorans* synthesizes the C_40_ carotenoids astaxanthin and lutein. The C_30_ carotenoid 4,4'-diaponeurosporene is produced by lactic acid bacteria (LAB) such as *Enterococcus* and *Lactiplantibacillus* [6-11]. Most microbial carotenoid studies focus on C_40_ carotenoids and there have been few reports on C_30_ carotenoid properties and production. The biosynthetic pathway of C_30_ carotenoids differs from that of C_40_ carotenoids and in LAB there are other carotenogenic genes such as dehydrosqualene desaturase (*crtN*) and dehydrosqualene synthase (*crtM*) which play vital roles in the conversion of farnesyl-pyrophosphate (FPP) to 4,4'-diaponeurosporene [12-16]. Microbial carotenoid synthesis can be improved by using specific microorganisms capable of inducing nutritional, physical, biochemical, and organoleptic modifications in a product or raw material. LAB can fill this role and be utilized not only as probiotics with health-promoting effects but also as carotenoid producers, particularly with their status as generally regarded as safe (GRAS) organisms. One of the species from the *Lactiplantibacillus* genus, *Lactiplantibacillus plantarum*, can synthesize a deep-yellow pigment identified as the C_30_ carotenoid 4,4'-diaponeurosporene, which is uncommon in nature [17-20].

There is currently limited research on the production of C_30_ carotenoids and the feasibility of industrial preparation of these compounds by scaling up production. Thus, our aim in this study was to determine the ideal conditions for optimal mass production of 4,4'-diaponeurosporene by *Lactiplantibacillus plantarum* subsp. *plantarum* KCCP11226^T^. To achieve this, culture conditions were optimized for sources essential to cell growth and metabolism, and stress was applied to promote carotenoid production. In addition, the effects of optimized factors on mass production of 4,4'-diaponeurosporene were determined by comparing production using shake-flasks and 5 L fermentors.

## Materials and Methods

### Chemicals

Yeast extract was purchased from Duchefa Biochemie (Haarlem, Netherlands). Beef extract, de Man Rogosa and Sharpe (MRS) and proteose peptone were purchased from KisanBio (MB Cell; Korea). Analytical-grade hexane and methanol were obtained from Samchun (Gyeonggi, Korea) and ThermoFisher (USA), respectively, and all other chemicals were of analytical grade and purchased from Sigma-Aldrich (USA).

### Bacterial Strain Growth and Expression

*Lactiplantibacillus plantarum* subsp. *plantarum* KCCP11226^T^, a previously isolated bacterial strain from kimchi (Korean traditional fermented food), was selected from among other strains for its high level of carotenoid production [20]. The strain was spread on de Man Rogosa and Sharpe (MRS) plates containing 1.5% agar or in MRS media at 30°C. For 4,4'-diaponeurosporene production, strains were cultured at 20°C with shaking (110 rpm). The composition of commercial MRS media used in the study was as follows: glucose (20 g/l), yeast extract (5 g/l), beef extract (10 g/l), proteose peptone (10 g/l), sodium acetate (5 g/l), tri-ammonium citrate (2 g/l), Tween 80 (1 g/l), dipotassium phosphate (2 g/l), magnesium sulfate (0.1 g/l) and manganese sulfate (0.05 g/l).

### Evaluation of Cell Growth and Carotenoid Concentration

The extraction of 4,4'-diaponeurosporene from *L. plantarum* subsp. *plantarum* KCCP11226^T^ was performed according to the method described by Hagi *et al*. [8] with modifications. Cultures of *L. plantarum* subsp. *plantarum* KCCP11226^T^ were grown at 30°C for 24 h with shaking (110 rpm) in 100 ml modified medium in an Erlenmeyer flask (250 ml). Cell growth was measured using a UV-Vis spectrophotometer (UV-1280, Shimadzu, Japan) at 600 nm. After 24 h, cells were harvested using a centrifuge (1580R, LaboGene, Denmark) at 10,000 ×*g* for 5 min. Ten milliliters of methanol were added to harvested cells, and the carotenoid was extracted from the resuspended pellet overnight at room temperature with an orbital shaker (R100, LaboGene, Denmark). After shaking, 5 ml distilled water and 10 ml hexane were added to the methanol extract. The extract was then agitated at room temperature for 2 h with an orbital shaker to transfer 4,4'-diaponeurosporene into the hexane supernatant. The carotenoid-containing supernatant was evaporated and resuspended in 1 ml petroleum ether. The amount of pigment was measured by UV-Vis spectrophotometry at A_470_. A time-course profile for cell density and carotenoid concentration was made by taking periodic measurements: every 2 h for 0 to 6 h; every 3 h for 6-15 h and 21-30 h; and at 48 h.

### Screening of Optimal Carbon and Nitrogen Sources

The effects of carbon and nitrogen sources on cell growth and 4,4'-diaponeurosporene production by *Lactiplantibacillus plantarum* subsp. *plantarum* KCCP11226^T^ were studied in modified MRS media. All carbon and nitrogen sources were screened using the One-Factor-at-A-Time (OFAT) method. Following overnight static incubation at 30°C in MRS medium, cultures of 100ml modified MRS media were prepared and agitated (110 rpm) at 30°C for 24 h. The carbon sources evaluated were: arabinose, fructose, galactose, glucose, lactose, maltose, mannitol, raffinose, sucrose and xylose. The other medium components were unaltered. The effect of nitrogen sources was assessed in modified MRS including 20 g/l of the selected optimal carbon source. The three components of modified MRS (yeast extract, beef extract and proteose peptone) were replaced by 25 g/l of the following nitrogen sources: beef extract, casamino acid, casitone, malt extract, peptone, proteose peptone, soytone, tryptone and yeast extract. Components other than carbon and nitrogen were maintained during the screening tests.

### Effects of Abiotic Stress on Carotenoid Production

The effects of abiotic stress on carotenoid production were determined by the variables of incubation temperature, initial pH, and salinity stress. Microbial cultures were grown in modified MRS containing optimized carbon and nitrogen sources, as well as a control of commercial MRS. The effect of temperature on carotenoid level was explored by culturing cells at 20, 25, 30 and 35°C. Cultures with 1% inoculation were grown at each temperature for 24 h in modified MRS with shaking. The effects of pH range on carotenoid level were evaluated on modified MRS adjusted to various pH values (pH 5.0–9.0 at intervals of 1.0 pH unit) using: HCl buffer pH 5.0–6.0 and Tris-base buffer pH 7.0–9.0. Salinity stress was evaluated in modified media containing NaCl concentrations between 0 and 8% (w/v) at 2% intervals. A time-course assay was performed using 1 L optimized medium, and cell density and carotenoid production were measured as before.

### Response Surface Methodology with Box-Behnken Design

Response surface methodology (RSM) was used to optimize the production of 4,4'-diaponeurosporene by *L. plantarum* subsp. *plantarum* KCCP11226^T^. The Box-Behnken design (BBD) was applied and three media variables, carbon source concentration, nitrogen source concentration and initial pH, were selected for optimization. As shown in Table S1, fifteen runs including three center points were performed. Each parameter was designated to three levels: high (1), medium (0), and low (-1). The predicted optimal values of the three parameters were calculated by a second-order polynomial:

$$Y=B_{0}+\sum B_{i}X_{i}+\sum B_{ii}X_{i}^{2}+\sum B_{ij}X_{i}X_{j}+\varepsilon ,$$

where *Y* (carotenoid concentration; A_470_/ml) is the dependent variable representing predicted response. The fitted response at the central point is represented by *B*_0_. *B*_i_, *B*_ii_, and *B*_ij_ which are the coefficients for linear, quadratic, and cross-product regression. The coded independent variables (*X*_1_ = carbon source concentration, *X*_2_ = nitrogen source concentration, *X*_3_ = initial pH) are represented by X_i_ and X_j_ (with *j* = *i* + 1) and random error (ε) is the measure of difference between observed and predicted values.

### Batch Fermentation

Following optimization, mass production of 4,4'-diaponeurosporene was performed in a lab-scale 5 L fermentor (Jar-Fermentor, Kobiotech, Korea) with optimized media. The seed culture was prepared by overnight static incubation at 30°C in 90 ml and 150 ml optimized media. Before inoculation, each seed culture was centrifuged at 10,000 ×*g* for 5min, and harvested cells were resuspended 30 ml medium. This was inoculated into 3 L of optimal modified medium in the 5 L fermentor at an inoculation ratio of 5% (v/v). Batch fermentation was conducted at a temperature of 25°C with 200 rpm, and aeration rates were set up at 0 (no aeration), 0.3, and 1.0 vvm to optimize the aeration rate in 5 L fermenter scale. Fermentation lasted 48 h, and periodic sampling was performed as before for the time-course profile. At each point of sampling, dissolved oxygen (DO) rate and pH were monitored, and subsequently, cell density and 4,4'-diaponeurosporene concentration were determined.

### Statistical Analysis

The GraphPad Prism 5.01 package (USA) was used for the visualization of results and analysis of variance (ANOVA) tests in this study. The significance between results was analyzed using one-way ANOVA and Tukey’s multiple comparisons test, with *p* < 0.05 considered significant. For RSM, the Box-Behnken design was performed on Minitab software 18.1 (USA) and the statistical significance of the factors and their interactions were confirmed by *t*-test and *p*-value parameters. The visualization of the predicted model was performed by R software 4.1.0 (Austria).

## Results and Discussion

### Cultivation of *L. plantarum* subsp. *plantarum* KCCP11226^T^ in Initial Conditions

The previous isolation of carotenoids from *L. plantarum* subsp. *plantarum* KCCP11226^T^ confirmed the identity of the C_30_ carotenoid as 4,4'-diaponeurosporene at 470 nm, along with the biosynthetic pathway that led to its production [21].

**Fig. 1:**
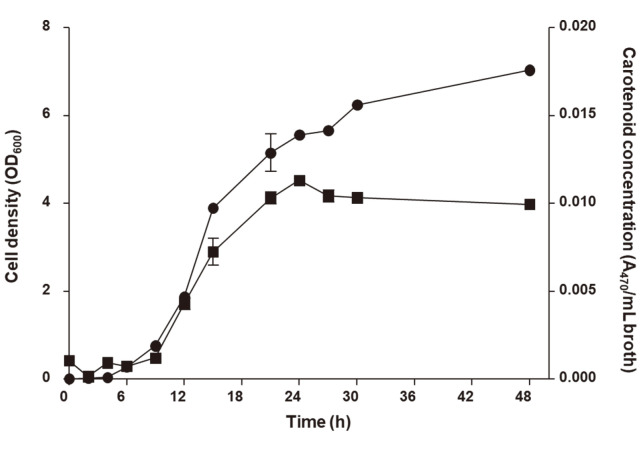
Time-course profile of cell density and carotenoid concentration from *L. plantarum* subsp. *plantarum* KCCP11226^T^ in flask containing commercial MRS medium at 30°C. Circles and squares indicate cell density and carotenoid concentration, respectively.

A time-course profile for cell density and carotenoid concentration during the growth of *L. plantarum* subsp. *plantarum* KCCP11226^T^ showed the results of the parameters when periodically measured. The trend of cell density and carotenoid concentration initially increased in a similar manner, while cell density increased until 48 h cultivation and the maximal carotenoid concentration was reached after 24 h of cultivation. Cells accumulated significantly higher carotenoid levels during their exponential growth phase and the highest cell density was measured after 48 h at 6.53 OD_600_, and the highest carotenoid concentration was 0.011 A_470_/ml at 24 h (Fig. 1).

### Optimization of Carbon and Nitrogen Sources

Carotenoid yields from biochemical pathways are influenced by substrate sources and composition, *i.e.*, physicochemical properties of the growth medium, such as pH and temperature; and controlled process conditions such as agitation and aeration [22]. Growth medium optimization for higher yields and biomass growth is regulated using components required for cell growth, such as amino acids, carbohydrates, peptides, and vitamins. Carbon and nitrogen are important for cell growth and energy supply, and are linked to secondary metabolism, metabolite productivity and product yield [23, 24].

**Fig. 2:**
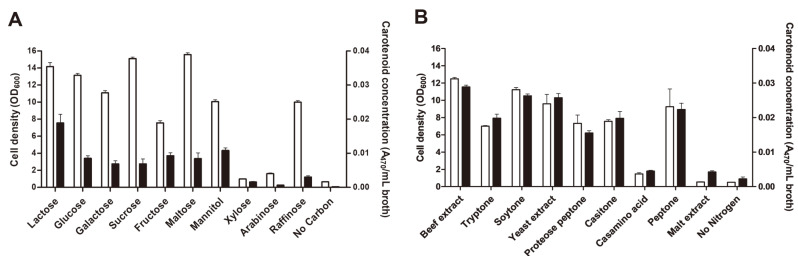
Effects of carbon sources (**A**) and nitrogen sources (**B**) on cell density and carotenoid concentration produced by *L. plantarum* subsp. *plantarum* KCCP11226^T^. Open and closed bars represent cell density and carotenoid concentration, respectively. *p < 0.05.

Cell growth and 4,4'-diaponeurosporene production were evaluated under various carbon and nitrogen sources in modified MRS (Fig. 2A). Among the carbon sources, maltose had the highest cell growth with an OD_600_ of 15.4, while sucrose and lactose showed significantly high cell growth with respective OD_600_ values of 15.3 and 14.4, meaning there were no significant differences between the three. Glucose, the original MRS carbon source, did not increase cell growth when compared to the other carbon sources. The lowest cell biomass was observed in the negative control (no carbon source) and pentoses arabinose and xylose, which consequently cannot have roles as proper energy sources for cell growth in *L. plantarum* subsp. *plantarum* KCCP11226^T^. Regarding carotenoid production, lactose exhibited the highest carotenoid concentration with 0.019 A_470_/ml. Apart from the pentoses and the negative control, which had near null production, and lactose which had the highest production, there was no significant variation between the other carbon sources, including trisaccharide raffinose. Therefore, lactose was selected as an optimal carbon source for both cell growth and 4,4'-diaponeurosporene production, which falls in line with lactose utilization preference as a typical property for the lactose metabolism pathway in LAB strains [25].

Modified MRS with lactose as the sole carbon source was used to study various nitrogen sources. Among them, beef extract proved to be optimal for cell growth and carotenoid production, with 12.6 OD_600_ and 0.029 A_470_/ml, respectively (Fig. 2B). Soytone and yeast extract also exhibited high cell density and carotenoid concentration and there was no significant difference between these and beef extract. The effect of nitrogen sources on concentration of biosynthetic products is dependent on species and strains. Carotenoid-producing microorganisms are typically fastidious and have complicated nutrient requirements due to their restricted ability to synthesize amino acids and vitamins. Organic nitrogen sources with nitrogen and specific growth factors are usually supplied to LAB for metabolite synthesis and can be ideal for carotenoid production, unlike inorganic nitrogen sources. As such, within selected media, organic complex nitrogen sources such as yeast and beef extracts are necessary for carotenoid production [26-28].

### Effect of Abiotic Stresses on Carotenoid Production

**Fig. 3:**
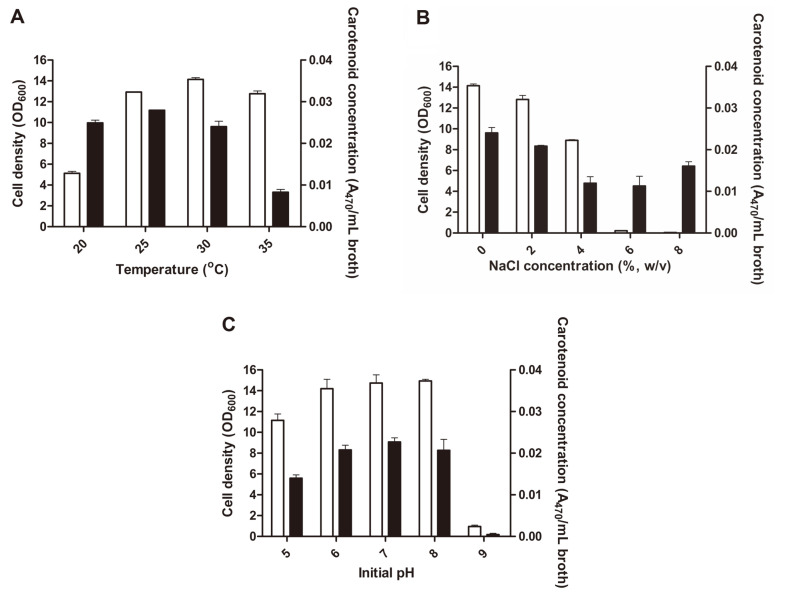
Effects of cultivation temperature (**A**), salinity stress (**B**), and initial pH (C) on cell density and carotenoid concentration produced by *L. plantarum* subsp. *plantarum* KCCP11226^T^. Open and closed bars represent cell density and carotenoid concentration, respectively.

The enhancement of microbial carotenoid synthesis and production yield can be accomplished by culture medium modifications and external stimulants. A number of studies have reported on the influence of abiotic stresses such as temperature, salinity stress, and pH on growth and synthesis capabilities [29, 30]. Temperature is a significant factor in carotenoid accumulation as it influences microbial growth, development, enzyme functionality and biosynthetic pathways. However, different strains have varying responses to temperature due to their specific optimal conditions and stress tolerances. In some cases, high temperatures result in greater yields of one carotenoid type and lower yields of another [31, 32]. In this study, similar to previous accounts on different LABs, the optimal temperature for cell density was 30°C (Fig. 3A) [33]. However, carotenoid production was highest at 25°C with a concentration of 0.028 A_470_/ml, which is consistent with reports that carotenoid accumulation is promoted at lower incubation temperatures [34]. Temperature conditions for high biomass yield are not necessarily related to enhanced carotenogenesis, and vice versa [31, 35].

Carotenoids are typically produced to protect the cell from stress and supplementation of growth media with NaCl for salinity stress has been found to promote carotenoid production in some microorganisms. The osmotic stress imposed by excessive salt concentrations is a known microbial stress factor and some species of bacteria can initiate carotenoid production following stress from induced reactive oxygen species (ROS) generation. Growth can be slowed by increased salt concentrations and if a microorganism is not suited to that environment or is unable to adapt or tolerate certain degrees of salinity, cell death rather than triggered antioxidant production is possible [36]. In this study, high salinity stress resulted in a decrease in cell density (Fig. 3B), and the strain was unable to grow in media with NaCl concentrations greater than 6%. The maximum 4,4'-diaponeurosporene concentration was shown in 0% NaCl with 0.024 A_470_/ml, and consequently, NaCl was not supplemented for optimal carotenoid production. However, carotenoid production increased with higher NaCl concentrations (over 4%) suggesting that salinity stress promoted 4,4'-diaponeurosporene production. These results corresponded with previous studies which reported that carotenoids were produced by microorganisms in stressful conditions as a cell-protecting mechanism [37].

Another stress factor employed for the optimization of 4,4'-diaponeurosporene production is the growth media pH which contributes to cell growth and carotenoid biosynthesis modulation [38]. Our study showed that cell density of *L. plantarum* subsp. *plantarum* KCCP11226^T^ increased as the initial pH increased from 5.0 to 8.0 but bacteria hardly grew at pH 9.0 with a sharp decline observed (Fig. 3C). The growth preference for cell biomass and carotenoid accumulation occurred at neutral pH rather than acidic or alkaline pH, which is supported by studies reporting that bacteria produce higher amounts of carotenoids in neutral or weak alkali media [39, 40]. The highest cell density was achieved at pH 8.0 (14.9) but there was no significant difference at pH 7.0 (14.7), which had the highest 4,4'-diaponeurosporene concentration of 0.023 A_470_/ml. The lowest biomass and carotenoid production occurred at pH 9.0, which was likely due to cell structure distortion, enzyme denaturation, and carotenoid instability under these conditions [38].

### Response Surface Methodology

**Fig. 4:**
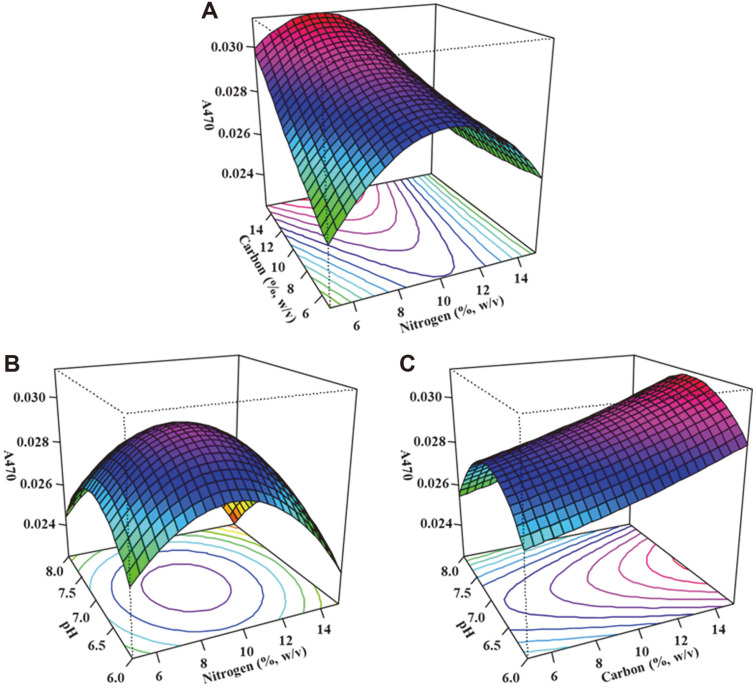
Three-dimensional response surface plots representing the effects of carbon and nitrogen concentrations (**A**), pH and nitrogen concentration (**B**), and pH and carbon concentrations (C) on predicted carotenoid concentration.

RSM was employed to study the influence of the selected variables and the optimal culture conditions were confirmed based on the BBD model. The three factors with the greatest influence on the production yield of 4,4'-diaponeurosporene were selected; lactose (optimal carbon source), beef extract (optimal nitrogen source), and initial pH of culture medium, and the actual results accurately followed the predicted values (Table 1). The results of the runs were analyzed by a quadratic regression model and the following equation was obtained:

$$Y=-0.0995+0.000760X_{1}+0.002875X_{2}+0.03213X_{3}-0.000127X_{2}^{2}-0.002333X_{3}^{2}-0.000052X_{1}X_{2}$$

where X_1_, X_2_ and X_3_ respectively represent lactose concentration, beef extract concentration and initial pH of culture medium. The model coefficient was calculated to be 0.869, indicating 86.9% of variability in the experiment with a good correlation between the experimental and predicted values [41]. The significance of the regression coefficient was analyzed by the ANOVA test (Table S2) and data analysis showed a significant effect (*p* < 0.05). The optimal levels of each factor, and the effects of interactions on carotenoid production as a function of the factors, were exhibited using three-dimensional response surface plots (Fig. 4).

Increasing the carbon content led to higher yields of carotenoids but nitrogen content was highest at 8.3% and showed decreased yields at 5 and 15% (w/v). A neutral pH was also observed as having greater ability to improve yields as the trend toward slightly acidic and slightly alkaline showed decreased 4,4'-diaponeurosporene production. From the RSM, the optimal conditions for 4,4'-diaponeurosporene production were: 15% (w/v) lactose, 8.3% (w/v) beef extract, and an initial pH of 6.9 for a predicted value of 0.032 A_470_/ml. The actual experimental value was 0.033 A_470_/ml, which was 1.06-fold that of the predicted value. Comparing the initial 4,4'-diaponeurosporene concentration in commercial MRS medium to that of optimized medium, there was a 3.3-fold increase in carotenoid production.

### Cultivation of *L. plantarum* subsp. *plantarum* KCCP11226^T^ in Optimal Conditions

**Fig. 5:**
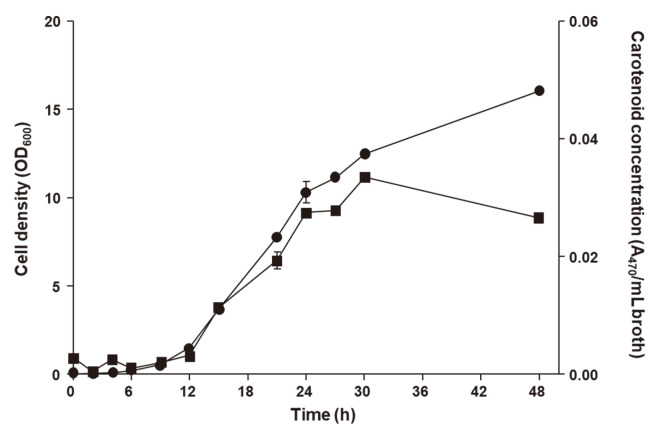
Time-course profile of cell density and carotenoid concentration from *L. plantarum* subsp. *plantarum* KCCP11226^T^ in flask containing modified optimal medium at 30°C. Circles and squares indicate cell density and carotenoid concentration, respectively.

To confirm the trend of cell density and 4,4'-diaponeurosporene production in the flask-scale cultivation with optimized conditions (15% (w/v) lactose, 8.3% (w/v) beef extract, and initial pH 6.9), a time-course assay was performed. One liter of optimized medium in a 2 L Erlenmeyer flask was inoculated with 1% seed culture and incubated at 25°C with agitation (110 rpm). Carotenoid concentration and cell density increased rapidly after 12 h and the trend remained similar up until 30h, at which point the highest carotenoid concentration of 0.034 A_470_/ml was recorded. Afterward, although cell density steadily increased, there was a decrease in carotenoid concentration (Fig. 5). The decrease in carotenoid concentration was likely due to degradation from increasing acidity and autooxidation. Neutral conditions are more ideal for carotenoid production and increasingly acidic environments exacerbate their degradation [42, 43].

### Batch Fermentation with Optimized Aeration Rate

**Fig. 6:**
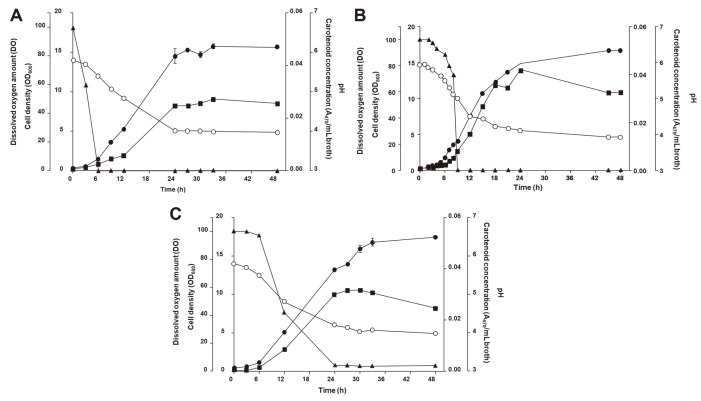
Time-course profiles of *L. plantarum* subsp. *plantarum* KCCP11226^T^ in lab-scale 5 L fermentor showing (**A**) no aeration, (**B**) 0.3 vvm aeration, and (C) 1 vvm aeration with corresponding cell density, 4,4'- diaponeurosporene concentration, pH and DO. Inoculation volume at 5% of fermentation medium; culture at 25°C with 200 rpm agitation. Closed circles, closed squares, open circles, and closed triangles indicate cell density, carotenoid concentration, pH, and DO, respectively.

Next, we investigated the aeration ratio in 5 L fermenter scale, because under oxygen-exposure conditions, LAB strains can enhance carotenoid production as a stress tolerance mechanism and response to aerobic conditions and oxidative stress [13, 44]. The time-course profile of *L. plantarum* subsp. *plantarum* KCCP11226^T^ cultured under different aeration rates of 0, 0.3 and 1 vvm confirmed their effects on carotenoid production (Fig. 6). In order to mimic flask cultivation conditions, *Lactiplantibacillus plantarum* subsp. *plantarum* KCCP11226^T^ was grown at 25°C with agitation (200 rpm) but no aeration. As shown in Fig. 6A, the trend of each factor was similar to that of the flask fermentations. However, the concentration of DO rapidly decreased at the start of the fermentation and reached 0 after only 6 h. The pH of the medium steadily decreased and at 3.98, was similar in value to previous flask cultures. Without aeration, the cell density and carotenoid concentration were significantly reduced. At zero aeration, the highest cell density and 4,4'-diaponeurosporene content occurred at 33 h with values of 15.8 and 0.030 A_470_/ml, respectively, both of which were much lower than flask culture and aerated fermentor production.

At 0.3 vvm, cell density and carotenoid accumulation slowly increased up to 6 h after which there was a sharp increase in both parameters (Fig. 6B). The increase in acidity followed the pattern of the 0 vvm fermentor and agitated flasks. Both cell density and 4,4'-diaponeurosporene content increased slowly up to 6 h after which they both increased sharply. There was a higher concentration of carotenoids after 24 h with 0.042 A_470_/ml; a 1.31-fold increase from flask cultivation. The pH decreased continuously with rising cell density to a final pH of 3.93 and cell density of 16.7 after 48 h. From 6-9 h, the DO rate rapidly decreased till it reached 0. Carotenoid production was highest at 24 h before decreasing gradually while cell density continued to increase at a slower rate after 24 h. The high cell density at 48 h indicated that accumulated carotenoids might be degraded during the log phase while the decrease in carotenoids during the stationary phase could be attributed to instability and degradation due to increased acidity from lactic acid production. At 0.3 vvm up until 24 h, the cell density and 4,4'-diaponeurosporene content generally rose in almost direct proportion, with the unexpected dip in the carotenoids at 20 h. This result was in accord with previous reports in which cell growth of *L. plantarum* species increased under aeration. When aeration conditions of 1 vvm were applied, the pH of the medium was similar to the previous tests, and there was a steady decline to 3.98 at the end of the fermentation (Fig. 6C). The DO rate also decreased as cell growth increased but the decrease was slower, with a steeper slope than that of 0.3 vvm. Once it reached 3-4, the DO rate was maintained until the end of the fermentation. In both 0 and 0.3 vvm, the DO rate completely decreased between 6 and 12 h, while at 1 vvm, the decrease was gradual from 6 to 24 h. The cell density was much higher under 1 vvm aeration and was 17.4 at the end of the fermentation showing that increased aeration enhanced cell growth.

The highest carotenoid concentration was 0.042 A_470_/ml broth at 24 h in 0.3 vvm aerated media, followed by 0.032 A_470_/ml broth at 30 h in 1 vvm aeration condition, indicating that while high levels of aeration volume can promote cell growth, this does not translate to increased carotenoid biosynthesis. Aeration was necessary for the enhancement of carotenoid production and controlling the rate of aeration can achieve mass production of 4,4'-diaponeurosporene. As such, 0.3 vvm was selected as the ideal aeration rate for optimal carotenoid production which, in a fermentor-culture, enhanced carotenoid production to a concentration of 0.042 A_470_/ml broth from that of 0.011 A_470_/ml broth when the strain was cultured in flasks. The highest carotenoid concentration from optimal fermentation conditions was 3.8-fold higher than carotenoid concentration when the strain was cultured at initial conditions in commercial MRS medium. This study demonstrates the possibility of mass production of 4,4'-diaponeurosporene by *L. plantarum* subsp. *plantarum* KCCP11226^T^ through the optimization of culture conditions and production scale-up from an Erlenmeyer flask to a lab-scale fermentor.

In this study we reported the enhancement of carotenoid 4,4'-diaponeurosporene production by *L. plantarum* subsp. *plantarum* KCCP11226^T^ through the optimization of culture conditions. Among the carbon and nitrogen sources for bacterial growth and carotenoid production, lactose and beef extract were the best sources, respectively. We also determined that the addition of abiotic stresses such as modifications to temperature, salinity, and pH of medium had an influence on carotenoid biosynthesis. Response surface methodology was used to optimize the factors of lactose and beef extract concentrations, as well as initial pH of medium. Optimization of carotenoid production was achieved in flask level using 15% lactose, 8.3% beef extract, 6.9 initial pH, and cultivation at 25°C. Under these conditions, the highest carotenoid concentration was 0.033 A_470_/ml broth, which was 3.3-fold higher than that of the initial condition. Optimization was further performed for upscale fermentation in a 5 L laboratory fermentor, with higher values achieved than in an Erlenmeyer flask culture. The maximized production of 4,4'-diaponeurosporene was 0.043 A_470_/ml broth and was obtained at 25°C with 200 rpm and 0.3 vvm in a fermentor. This value was 4.2-fold that of the initial unoptimized culture conditions, and 1.3-fold that of optimized conditions, respectively. The results from this research showed that scaling up production for industrial application is feasible with modifications made to the physical and chemical factors that influence the bacterial growth and biosynthetic capabilities of *L. plantarum* subsp. *plantarum* KCCP11226^T^. Increasing the yield further can be achieved through recombinant technology by mutation or evolution in order to obtain a more adaptable microorganism with enhanced performance for the preferred compound.

## Supplemental Materials

Supplementary data for this paper are available on-line only at http://jmb.or.kr.

## Figures and Tables

**Table 1:** Experimental Box-Behnken design and response actual value and predicted value matrix.

Standard	X_1_	X_2_	X_3_	Response	

	Carbon source (%, w/v)	Nitrogen source (%, w/v)	pH	Carotenoid concentration (A_470_/ml)	

				Actual value	Predicted value
1	5	5	7	0.024	0.025
2	15	5	7	0.029	0.030
3	5	15	7	0.029	0.027
4	15	15	7	0.027	0.026
5	5	10	6	0.027	0.027
6	15	10	6	0.029	0.029
7	5	10	8	0.026	0.026
8	15	10	8	0.028	0.028
9	10	5	6	0.027	0.025
10	10	15	6	0.023	0.024
11	10	5	8	0.025	0.024
12	10	15	8	0.021	0.023
13	10	10	7	0.030	0.029
14	10	10	7	0.030	0.029
15	10	10	7	0.028	0.029
