# Supplementary material for: Enhanced Production of C30 Carotenoid 4,4'-Diaponeurosporene by Optimizing Culture Conditions of Lactiplantibacillus plantarum subsp. plantarum KCCP11226T
Source: J Microbiol Biotechnol. 2022 May 31;32(7):892–901. doi: 10.4014/jmb.2204.04035 (PMC9628921; doi:10.4014/jmb.2204.04035)
Supplement: Supplementary file 1 [file jmb-32-7-892-supple.pdf]

J. Microbiol. Biotechnol.
https://doi.org/10.4014/jmb.2204.04035

Supplementary Table S1. Box-Behnken design for evaluation of carotenoid production from *L. plantarum* subsp. *plantarum* KCCP11226^{T} with coded factors and values.

Standard	X_1	X_2	X_3
	Carbon source (%, w/v)	Nitrogen source (%, w/v)	pH
1	-1 (5)	-1 (5)	0 (7)
2	1 (15)	-1 (5)	0 (7)
3	-1 (5)	1 (15)	0 (7)
4	1 (15)	1 (15)	0 (7)
5	-1 (5)	0 (10)	-1 (6)
6	1 (15)	0 (10)	-1 (6)
7	-1 (5)	0 (10)	1 (8)
8	1 (15)	0 (10)	1 (8)
9	0 (10)	-1 (5)	-1 (6)
10	0 (10)	1 (15)	-1 (6)
11	0 (10)	-1 (5)	1 (8)
12	0 (10)	1 (15)	1 (8)
13	0 (10)	0 (10)	0 (7)
14	0 (10)	0 (10)	0 (7)
15	0 (10)	0 (10)	0 (7)

Supplementary Table S2. Analysis of variance for experimental model.

Source	DF	Adj SS	Adj MS	F-value	P-value Prob > F	Remarks
Model	6	0.000081	0.000013	8.84	< 0.05	Significant
A	1	0.000012	0.000012	7.64	< 0.05	
B	1	0.000006	0.000006	4.17	0.075	
C	1	0.000002	0.000002	1.53	0.252	
B^2	1	0.000037	0.000037	24.42	< 0.05	
C^2	1	0.000020	0.000020	13.23	< 0.05	
AB	1	0.000007	0.000007	4.41	0.069	
Lack of fit	6	0.000010	0.000002	1.67	0.421	
Pure error	2	0.000002	0.000001			
Residual	14	0.000093				Not significant
